# Anticipated vs. Experienced Pain at Site of Spinal Needle Insertion in Patients Undergoing Elective Lower Segment Caesarean Section: Perspective from Resource-Limited Region

**DOI:** 10.1155/2023/5516346

**Published:** 2023-06-20

**Authors:** Waleed Bin Ghaffar, Fauzia Minai

**Affiliations:** Aga Khan University Hospital, Karachi, Pakistan

## Abstract

**Background:**

Despite significant advantages, approximately 20% of pregnant patients refuse spinal anaesthesia in caesarean section due to fear of spinal needle prick. Studies have shown that the patient's expectation of pain is higher than what they experience in real. The objective was to evaluate the difference between anticipated and actually experienced pain at the spinal needle insertion site in spinal anaesthesia for pregnant women undergoing elective lower segment caesarean section (ELSCS).

**Method:**

The cross-sectional study was conducted in a labour room suite of a tertiary care hospital.

**Results:**

A total of 50 patients scheduled for ELSCS were included. The median experienced pain at the site of spinal needle insertion was significantly low as compared to anticipated pain (*P* value < 0.01). For the identification of predictors impacting the anticipated and experienced pain, univariate and multivariate regression models were applied. Amsterdam Preoperative Anxiety and Information Scale ≥11 for anticipated pain showed a statistically significant positive correlation in univariate (coefficient: 2.59; 95% CI: 1.49 to 3.68; *P* value < 0.001) and multivariable analyses (coefficient: 2.51; 95% CI: 1.36 to 3.67; *P* value < 0.001). Thus, anxiety was associated with statistically significant higher anticipated pain.

**Conclusion:**

In conclusion, there is a remarkable difference in the obstetric population between anticipated and actually experienced pain at the site of spinal needle insertion in ELSCS.

## 1. Introduction

A caesarean section (CS) is the birth of an infant through incisions in the abdomen (laparotomy) and uterus (hysterectomy) when natural childbirth is likely to cause harm to either the mother or fetus or both [1]. Since the last decade, the trend of CS has greatly increased. According to data from 169 countries, births via CS have doubled over a span of 15 years [1]. In Pakistan, the rate of CS deliveries has upsurged from 3.2% in 1990 to 19.6% in 2018 [2].

Globally, spinal anaesthesia (SA) is regarded as a preferred technique in conducting CS [3]. In comparison to general anaesthesia (GA), there is decreased postoperative morbidity and mortality in patients who underwent CS under SA [4]. Limited data from the Pakistani population showed a higher patient satisfaction rate with SA [5]. Despite significant advantages, some patients refuse SA when offered as a choice. This happens due to anxiety of needle pain, fear of postregional backache, inadequate anaesthesia, perioperative discomfort, intraoperative awareness, previous bad experience, concerns of paralysis, postdural puncture headache, and resistance from surgeon or family [6, 7]. Fear of needles is common among patients in general as well as pregnant patients [8]. A study conducted by Rahee et al. showed that 15.9% of pregnant patients were dissatisfied due to needle pain with SA [9]. Similarly, in the Pakistani population, 20.1% of pregnant patients refused SA due to the fear of spinal needle pain at the insertion site [10].

Studies have shown that the patient's expectation of pain is higher than what they experience in real. Yano et al. studied predicted and perceived pain in females undergoing elective CS and found overestimated anticipated pain by patients [11]. Mogensen et al. also found higher expected pain in the epidural procedure [12]. Nonetheless, Asian countries pose different cultural, educational, and socioeconomic status. As stated above, in the South Asian region, more females tend to favour GA due to fear of spinal needle pain [10].

Our primary objective was to check the null hypothesis that there was no difference between anticipated and experienced pain at the spinal needle insertion site due to the conduct of spinal anaesthesia for pregnant women undergoing elective lower segment CS.

Our secondary objective was to analyse the relationship between preoperative anxiety and pain (both anticipated and experienced) at the site of spinal needle insertion in the same patients.

## 2. Materials and Methods

The study was approved from the Institutional Ethics Committee of Aga Khan University Hospital (ERC number 2019-1271-3245). The Aga Khan University Hospital is a tertiary care hospital with a dedicated labour suite which comprises 12 labour rooms, 1 preoperative room, and 2 operating rooms. From 1st April 2019 to 30 September 2019, 688 elective CS were done there. This cross-sectional study was conducted in the labour room suite.

The patients with age ≥18 with scheduled elective CS were included. The exclusion criteria were emergency CS, refusal to take part in the study, inability to comprehend the information, patients with contraindication to SA, previous SA experience, patients with a history of chronic back pain, more than two attempts for SA, duration of spinal conduct more than 20 min, and use of cutting spinal needle and its size other than 25 G. The duration of spinal conduct was considered as the time taken from start of skin preparation to withdrawal of the needle. An attempt was defined as from insertion of the introducer needle and spinal needle till complete withdrawal from the skin [13].

Written and informed consent of all patients was taken by the primary researcher who was enrolled in the study as per inclusion criteria. Before the surgical procedure, selected patients were evaluated for preoperative anxiety.

For the assessment of anxiety, Amsterdam Preoperative Anxiety and Information Scale (APAIS) was used. The APAIS is a short and reliable tool for the assessment of preoperative anxiety, in which the respondent answers from the 6 items [14].

The patients were informed in detail regarding the conduct of spinal anaesthesia. They explained possible discomfort due to the process of identification of intervertebral space and pain because of local anaesthetic infiltration. They were given a detailed description of spinal anaesthesia including the use of a 25 G needle for local infiltration followed by the insertion of a 20 G introducer needle and 25 G spinal needle (Pencan). All patients were informed regarding the possibility of pain despite local anaesthetic infiltration during introducer and spinal needle insertion and the possibility of more local anaesthesia administration at that site. The Numerical Pain Rating Scale (NPRS) was explained, and the same scale was used to assess anticipated pain by the primary investigator in each patient [15].

None of the patients was given any preoperative anxiolytics. In all patients, spinal anaesthesia was performed by a consultant anaesthesiologist or a resident with a minimum of 2 years of experience. A 20 G intravenous cannula was maintained in the preoperative area. In the operating room after the application of standard monitoring, crystalloid (Ringer's lactate solution or 0.9% normal saline) was administered at 15–20 ml/kg. All patients were positioned in sitting posture and identification of landmarks was done, i.e., third and fourth lumbar vertebral interspace or fourth and fifth lumber intervertebral space. Aseptic measures were practiced including the application of 2% chlorhexidine alcohol antiseptic. The skin and the subsequent layer were infiltrated via a 25 G needle with 2% lignocaine with dose calculation as per body weight (3-4 mg/kg). After the application of local infiltration, a standard 45 second interval was taken to establish the effect of local anaesthetic. Then, with the help of a 20 G introducer needle, the dura layer was pierced with a pencil point 25 G needle (Pencan). After confirmation of free flow of cerebrospinal fluid, 0.5% bupivacaine 10 to 14 mg and fentanyl 10 to 15 micrograms were injected. After completion of spinal anaesthesia, patients were placed supine on the operating table and asked about the intensity of pain experienced as per the NPRS by the primary investigator. The remainder of the procedure was carried out as usual. The segment of the spinal block was confirmed with the application of an ice-cold pack, and if adequate, surgery was commenced. Postoperatively, patients were shifted to the postanaesthesia care unit and the reversal of the spinal block was assessed.

## 3. Statistical Analysis

STATA version 12.0 was used to compute the sample size for the study proposed by Mogensen et al. which reported that the median anticipated and experienced pain score was 12 (effect size 0.52). At a significance level of 0.05, 42 patients were required to achieve 90% power to detect a 20% difference in numerical pain scale between anticipated and experienced pain. We enrolled 50 patients with adjusting for the 15% dropout.

Data were analysed by Statistical Package for the Social Sciences version 19 (SPSS Inc., Chicago, IL). The Kolmogorov–Smirnov test was applied to observe the normality of the outcome which is the pain score. It turned out that numerical variables showed asymmetric behavior. Therefore, for numerical variables, we computed the median with the first and third quartiles. The categorical variables such as comorbid, number of attempts, duration of spinal conduct, and level of anaesthesiologist were computed as frequency and percentage. We applied the Wilcoxon rank-sum test and calculated the effect size (pseudomedian difference) and the 95% confidence interval, in order to determine the difference between anticipated and experienced median pain scores. Simple regression univariate and multivariable analyses were performed to model the pain score (anticipated and experienced) on the basis of predictors. *P* ≤ 0.05 was considered statistically significant.

## 4. Results

A total of 50 women fulfilling the inclusion criteria were recruited in this study. The median age was 29 years (min, max: 19–42). Most patients underwent spinal anaesthesia in the first attempt (88%). The duration of spinal conduct was also within 10 minutes, in a majority (86%) of cases. SA was performed mostly by the residents (80%). The rest of the details can be seen in Table 1.

**Figure 1:**
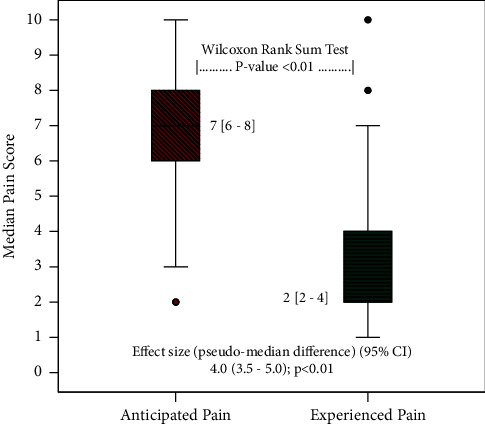
Comparison of anticipated and experienced pain score.

Figure 1 depicts the comparison of anticipated and experienced pain scores in these patients. The experienced pain score at the site of spinal needle insertion was significantly low as compared to anticipated pain (median anticipated pain 7; median experienced pain 2; *P* value <0.01). The pseudomedian difference was also statistically significant (pseudomedian difference 4; *P* value <0.05)

Table 2 shows the comparison of anticipated and experienced pain with the age, number of attempts, duration of the procedure, and the designation of an anaesthesiologist. In patients with a successful single attempt, there was a statistically significant difference (*P* value 0.0005) between the anticipated and experienced pain in comparison to the patients in whom a second attempt was made (*P* value 0.074). In regard to the designation of the anaesthesiologist (residents vs. consultants), the median anticipated pain was the same; however, the median experienced pain was lower among the patients in whom the resident placed the spinal anaesthesia. Other variables including age groups, duration of spinal conduct, and APAIS score showed no difference between the subjects.

**Figure 2:**
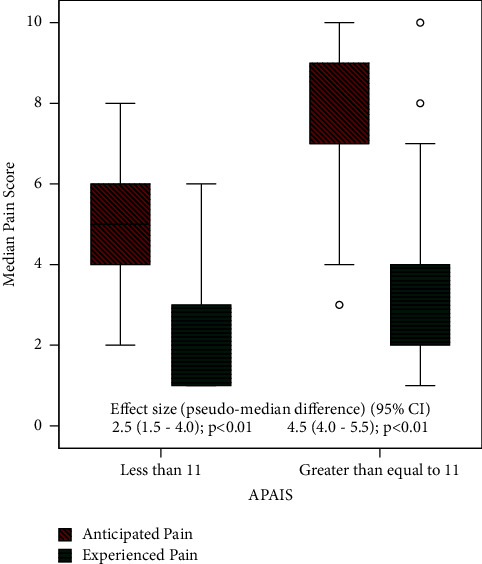
Comparison of anticipated and experienced pain score in relation to preoperative anxiety.

As shown in Figure 2, the pain was also assessed in relation to anxiety. For preoperative anxiety, APAIS was used. The APAIS cutoff of 11 has good sensitivity for the identification of preoperative anxiety [14]. The pseudomedian difference in APAIS <11 was 2.5 (*P* value <0.01) and APAIS >11 was 4.5 (*P* value <0.01). The patients with APAIS <11 had lower anticipated and experienced median pain scores in comparison to APAIS ≥11. This illustrates that anxiety had a positive correlation with the severity of pain. In both populations, the anticipated pain was higher than the experienced pain.

A univariate and multivariable regression model was applied to identify the predictors associated with the anticipated and experienced pain. In regard to anticipated pain, the APAIS ≥11 showed a significantly positive correlation in univariate analysis (coefficient: 2.59; 95% CI: 1.49 to 3.68; *P* value < 0.001) and multivariable analysis (coefficient: 2.51; 95% CI: 1.36 to 3.67; *P* value < 0.001). Thus, in patients with increased anxiety, the anticipated pain was higher. There was also a negative correlation of pain when SA was conducted by the residents in comparison to consultants, further verifying our previous finding; however, the difference was not statistically significant in both univariate (coefficient: 0.44; 95% CI: 1.06 to 1.95; *P* value 0.557) and multivariable analyses (coefficient: 0.433; 95% CI: −0.87 to 1.74; *P* value 0.509). The detail of other variables can be seen in Table 3.

The key predictors of experienced pain in Table 4 were the same as anticipated pain (Table 3). The patients with APAIS ≥11 experienced more pain; however, there was no statistical significance in the univariate analysis (coefficient: 1.04; 95% CI: −0.26 to 2.35; *P* value 0.115) and multivariable analysis (coefficient: 0.98; 95% CI: −0.41 to 2.38; *P* value 0.163). This shows that APAIS ≥11 was linked more with anticipated pain than experienced pain. The SA by the residents also showed a negative correlation with experienced pain univariate analysis (coefficient: 0.009; 95% CI: −1.43 to 1.62; *P* value 0.899) and multivariable analysis (coefficient: 0.98; 95% CI: −0.41 to 2.38; *P* value 0.163).

## 5. Discussion

It is acknowledged that anticipated pain is greater in comparison to actually experienced pain [8, 11, 12, 16, 17]. Nonetheless, there are very scarce data regarding anticipated vs. actually experienced pain due to the conduct of spinal anaesthesia in pregnant patients undergoing CS. In a previous study in females undergoing elective CS, overall predicted pain was 1.7-fold higher (*P* < 0.001) for epidural anaesthesia and 1.2-fold more (*P*=0.031) for spinal anaesthesia as compared to perceived pain [11]. Mogensen et al. reported similar outcomes in epidural anaesthesia in patients undergoing elective major abdominal surgery. The median expected pain was 5 and the median experienced pain was 2 (*P* < 0.0001) [12]. Our study further confirmed previous findings. The results of our study showed the anticipated median spinal needle pain score was significantly high as compared to the experienced mean spinal needle pain score (7 vs. 2; *P* < 0.01). In terms of NPRS, the anticipated pain in our study was moderate to severe, while the actually experienced pain was classified in the mild category.

We included only those patients who underwent spinal anaesthesia with a pencil point 25 G needle along with a 20 G introducer needle. Thus, local infiltration was necessary. Therefore, we measured the anticipated pain of the whole procedure of the spinal block, i.e., from the position and local infiltration until the application of the block. This is in contrast to T. Yano et al. who did not apply local anaesthetic infiltration for the spinal block and measured only spinal needle pain [11]. However, Mogensen et al. applied local anaesthetic infiltration before 18 G Tuohy needle insertion and measured pain due to an epidural catheter [12]. We used NPRS to assess pain. A systematic review, comparing NRS, VAS, and VRS, revealed that NRS had a higher compliance rate and was simple to use [15].

All of the attempted spinal blocks were successful in our study. In our opinion, success rate was due to thorough preoperative counselling and a detailed description of the procedure. Moreover, the spinal block was performed only in the sitting position with the median approach. The paramedian approach is an independent predictor of failed spinal and lateral positions associated with considerable difficulty [18, 19]. As per demographics, patients included by T. Yano et al. had an average mean age of 31 ± 5.1. These patients underwent epidural puncture and then afterwards spinal puncture. In comparison to epidural puncture, the mean difference between predicted and perceived pain was much lower in the spinal puncture [11]. The average median age of patients in our study was comparable, i.e., 29 years (19–42). However, the difference between anticipated and actually experienced pain was significantly higher, i.e., 5. This difference was due to decreased experienced pain in our study. In our opinion, this difference can be due to the use of local anaesthetic infiltration before the use of the spinal needle. In the literature, patients have reported less pain with local anaesthetic infiltration before epidural puncture. T Yano also proposed slow local infiltration to decrease perceived spinal puncture pain [11].

Rhee et al., while identifying factors in patient dissatisfaction and refusal for spinal anaesthesia, revealed that spinal puncture attempts of greater than 3 would result in a dissatisfaction rate and refusal rate of 9.5% and 5.7%, respectively [9]. In regards to the number of attempts, only 6 patients in our study underwent 2nd attempts. However, among both groups, the experienced pain was the same. We noticed that the duration of the procedure was associated with both decreased experience of pain and the increased difference between anticipated and actually experienced pain. No previous studies have compared this factor to experience pain during the procedure of spinal anaesthesia.

There was no correlation between pain with the seniority of anaesthesiologist performing the block. Although the anticipated pain was the same from both groups (resident vs. consultants) yet actually experienced pain was less in the resident group. Our hospital is a university teaching hospital. The residents go through a stringent training pathway. Thus, consultants supervise the trainees, and residents are the ones who usually perform the procedure. This can be a possible reason for decreased experienced pain in patients in which residents performed the SA. A similar finding was also observed by Ružman et al. and Atallah et al., who revealed that spinal anaesthesia performed by younger residents was associated with a more first-attempt success rate [18, 19].

We recommend that providing patients with psychological support and thorough information regarding the procedure may help not only to alleviate their needle pain anxiety, but also facilitate consent for spinal anaesthesia. The provision of an information brochure regarding spinal anaesthesia can be a useful adjunct. Many approaches are used to decrease pain at the site of spinal needle insertion, e.g., application of an EMLA (eutectic mixture of a local anaesthetic) patch, slow infiltration of local anaesthesia in the subcutaneous tissue [20].

Preoperative anxiety is an independent predictor of increased pain [21]. Patients usually suffer the highest fear during the preparation and administration of the neuraxial block [22]. Pregnant patients undergoing LSCS may show overstated pain in comparison to other populations. Thus, these results can be different in other patient populations. We found a strong correlation between anxiety with the pain (anticipated and experienced). The higher anxiety score as per APAIS was in correlation with the increased pain.

Regarding the limitations of our study, spinal anaesthesia was not performed by one anaesthesiologist. Although the number of spinal blocks applied in the first attempt was considerably high, it shows that all anaesthetists were competent enough regarding experience and the number of performed procedures. However, it can be a limitation as needle handling varies from person to person. The result might have been different if only one anaesthesiologist had performed all spinal blocks. Another limitation was that we were unable to standardize the speed and the pressure of local anaesthetic injection in the subcutaneous tissue. Patients' pain perception can vary with the speed and pressure of the local anaesthetic injection [23, 24]. The slow injection helps to minimize pain [23]. In addition, to decrease pain and anxiety, the recommendation is to inject a local anaesthetic under low pressure (less than 306 mmHg). It may also affect the outcome of experienced pain.

## 6. Conclusion

In conclusion, there is a remarkable difference in the obstetric population between anticipated and actually experienced pain while performing spinal anaesthesia. Based on this study's results, we can counsel the patients about the procedural pain during spinal anaesthesia. This will lead to a less refusal rate of spinal anaesthesia CS. However, further research is needed in a large population group.

## Figures and Tables

**Table 1:** Characteristics of patients.

Variables	Point estimates
Age (Years)	29 (19–42)

*Comorbidities*	
Hypertension	6 (12%)
Diabetic	10 (20%)
Anemia	6 (12%)
Obese	3 (6%)
Others	4 (8%)

*No. of attempts*	
One	44 (88%)
Two	6 (12%)

*Duration of spinal conduct*	
Within 10 minutes	43 (86%)
11 to 15 minutes	7 (14%)

*Designation of anesthesiologist*	
Resident	40 (80%)
Consultant	10 (20%)

**Table 2:** Comparison of anticipated and experienced pain score.

Variables in categories	Anticipated (E1)	Experienced (E2)	^*∗*^ *P* value
*Age groups*			
≤30 Years (*n* = 29)	7 (5–8)	3 (2–5)	<0.001
>30 Years (*n* = 21)	7 (6–8)	2 (2–4)	0.001

*Number of attempts*			
1 (*n* = 44)	7 (6–8)	2 (2–4)	<0.001
2 (*n* = 6)	7 (4–7)	2 (2–4)	0.074

*Duration of spinal conduct*			
≤10 min	7 (6–8)	2 (2–4)	<0.001
>10 min	8 (7–9)	3 (2–7)	0.042

*Designation of anesthesiologist*			
Resident	7 (6–8)	2 (2–4)	<0.001
Consultant	7 (5–8)	3 (2–4)	0.107

*APAIS score*			
<11 (*n* = 13)	5 (4–6)	2 (1–3)	0.001
≥11 (*n* = 37)	7 (7–9)	3 (2–4)	<0.001

Data are presented as median (25th–75th percentile). ^*∗*^Wilcoxon signed-rank test.

**Table 3:** Predictors associated with anticipated pain.

Variables	Univariate analysis		Multivariable analysis	
	Coefficient (95% CI)	*P* Value	Coefficient (95% CI)	*P* value
Age	0.01 (−0.09, 0.10)	0.913	−0.01 (−0.09, 0.07)	0.792

*Number of attempts*				
1 attempt	1.0 (reference)			
>1 attempt	−0.66 (−2.44, 1.11)	0.456	−1.46 (−3.30, 0.39)	0.119

*Duration of spinal*				
≤10 min	1.0 (reference)			
>10 min	0.75 (−0.90, 2.42)	0.365	0.75 (−1.00, 2.50)	0.391

*Designation of anaesthesiologist*				
Consultant	1.0 (reference)			
Resident	0.44 (1.06, 1.95)	0.557	0.433 (−0.87, 1.74)	0.509

*APAIS*				
<11	1.0 (reference)			
≥11	2.59 (1.49, 3.68)	<0.001	2.51 (1.36, 3.67)	<0.001

**Table 4:** Predictors associated with experienced pain.

Variables	Univariate analysis		Multivariable analysis	
	Coefficient (95% CI)	*P* value	Coefficient (95% CI)	*P* value
Age	−0.04 (−0.13, 0.05)	0.389	−0.05 (−0.15, 0.04)	0.315

*Number of attempts*				
1 attempt	1.0 (reference)			
>1 attempt	0.09 (−1.71, 1.90)	0.913	−0.37 (−2.61, 1.86)	0.737

*Duration of spinal*				
≤10 min	1.0 (reference)			
>10 min	0.73 (−0.94, 2.42)	0.383	0.68 (−1.44, 2.80)	0.520

*Designation of anaesthesiologist*				
Consultant	1.0 (reference)			
Resident	0.09 (−1.43, 1.62)	0.899	0.16 (−1.42, 1.75)	0.833

*APAIS*				
<11	1.0 (reference)			
≥11	1.04 (−0.26, 2.35)	0.115	0.98 (−0.41, 2.38)	0.163

## Data Availability

The data used to support the findings of this study have not been made available because the institution's medical record cannot be shared.
